# Early Post-natal Care Services Utilization and its associated factors among mothers Systemic Review and Meta-Analysis^☆^

**DOI:** 10.1016/j.heliyon.2023.e23760

**Published:** 2023-12-18

**Authors:** Tigist Seid Yimer, Fillorenes Ayalew Sisay, Habtamu Gebrehana Belay, Gedefaye Nibret Mihretie, Eyaya Habtie Dagnaw, Wassie Yazie Ferede

**Affiliations:** Department of Midwifery College of Medicine and Health Sciences, Debre Tabor University, Ethiopia

**Keywords:** Early post-natal care services, Prevalence, Associated factors, Systematic review, meta-Analysis, Utilization, Ethiopia

## Abstract

**Background:**

Early Post-natal Care Services are defined as the care given to the mother and the newborn baby after childbirth of a few weeks. This time is the most life-threatening time since most maternal and neonatal mortality takes place.

**Methods:**

The preferred reporting elements for the Systematic Review and Meta-analysis (PRISMA) checklist methodology were used to build the current systematic review and meta-analysis. A systematic literature search was done at the electronic database using the Pub Med database, PubMed/MED-LINE, CINAHL, Google Scholar, Google, Web of Sciences, and Google Scholar to identify potential research. The retrieved author/year, study region, design, and sample size of all the authors. A standardized data-gathering measuring tool was used to obtain the data. I^2^ test statics were used to verify the heterogeneity among the investigations. The statistical program STATA 17 was used to analyze the data. Analysis of sensitivity was verified. The asymmetry of the funnel plot and statistically significant Egger's test at a 5 % significant level indicated the presence of publication bias. The pooled prevalence of mothers' use of early postnatal care services and the factors associated with it were determined using a random effect model.

**Result:**

A total of 4498 mothers were involved in 10 studies. The pooled prevalence of Early Postnatal Care Services Utilization and Its Associated Factors among Mothers in Ethiopia was (28.51 % (95%CI, [20.95, 36.06]). According to the pooled effect, utilizing postnatal care services early was associated with formal education four points, or seven times (OR = 4.73 (95%CI, 3.12, 7.18)) higher likelihood. An early postnatal care service user is three times more likely to know early post-care visits (OR = 3.63 (95%CI, 1.25, and 10.50)).

Early postnatal care service consumption is five times more likely to be associated with birth complications (OR = 4.93 (95%CI, 2.62, 9.27)). Being three times more likely to use early post-natal care services if an ANC is present (OR = 3.56 (95%CI, 2.03, 6.26)). Women who had traveled fewer than 2 h were three times as likely to have used early post-natal care services (OR = 3.47 (95%CI, 2.32, 5.20)). Early Post-natal care services utilization history ((OR = 2.26 (95%CI, 1.68, 3.04)) women who had previously used early post-natal care services.

**Conclusion and recommendation:**

In comparison to national guidelines, the WHO, and other research, Ethiopia's pooled prevalence of accessing early postnatal care services is low. Prenatal care service use and birth complications also have a significant impact on the use of early postnatal care services. Improving early postnatal care service usage requires expanding the availability of antenatal care services on a national scale. Strengthening prenatal care services, increasing the number of health centers and health posts, increasing delivery at health facilities, and emphasizing or improving mothers' knowledge of and attitudes toward early post-natal care contact are all critical to improving quality of life and lowering neonatal and maternal morbidity and mortality. Future studies and the Ethiopian Ministry of Health should concentrate on improving the use of prenatal care services, minimizing and managing birth complications, and enhancing the use of early postnatal care services.

## Plain English summary

Early Post-natal Care Services are defined as the period after childbirth of a few weeks. The WHO recommended a total of 4 contacts. The first contacts are recommended on day 3 (48–72hr). The key purpose of the contacts is to detect any maternal and neonatal complications and treat them. This period is the most life-threatening time because most maternal and neonatal mortality takes place, more than 50 % of post-natal maternal deaths occur within the first 24 h and more than 66 % of deaths occur within the first week.

To identify potential studies, use the Pub Med database, PubMed/MED-LINE, CINAHL, Google Scholar Google, Web of Sciences, and Google Scholar electronic database. Unpublished studies were also updated from library and research center sources. Based on study eligibility criteria, a total of 10 primary studies evaluating the utilization of early postnatal care services and its associated factors were included.

This systematic review and meta-analysis establish that the pooled prevalence of Early Post-natal Care Services Utilization and Its Associated Factors among women in Ethiopia was 28.51 %. Educational status (having formal education), knowledge about early postnatal care visits, birth complication, institutional delivery, history of ANC follow-up distance from the health center, women's decision-making empowerment, and previous history of early post-natal care contacts were recognized as Early Post-natal Care Services Utilization in Ethiopia.

The pooled prevalence of Early Post-natal Care Services Utilization in Ethiopia is low when compared with different studies, the WHO, and national recommendations. Therefore, emphasizing or improving the knowledge and attitudes of the mothers towards early postnatal care visits, boosting delivery at health facility increasing the number of Health centers, and health posts are essential to improve quality of life and diminish neonatal and maternal morbidity and mortality.

## 1 Introduction

Post-natal care is the care given to the mother and her newborn baby directly after birth and for the first six weeks of life [1]. WHO suggested a minimum of four post-natal care contacts. The first two weeks after birth are a crucial time to encourage health, ascertain health problems, and support the transition to well-women and well-infant care. In addition to this Effective and safe clinical and non-clinical interventions, as well as health systems and health promotion interventions for vital care during the post-natal period, advance the excellence of post-natal care for women and newborns [2].

The post-natal period is the most life-threatening period since the greatest maternal and neonatal mortality takes place, around 50 % of post-natal maternal demise happens within the first 24 h And more than 66 % of deaths happen within the first week [3]. Universally in 2017, due to complications going on throughout the pregnancy, childbirth, and post-natal times every year nearby 29, 5000 women globally miss their lives in the world, 94 % of which are from developing countries. Sub-Saharan Africa and Southern Asia accounted for nearly 86 % (254,000) of the expected universal maternal demises. In this number, sub-Saharan Africa alone is responsible for nearly two-thirds or 66 % (196,000) of those deaths [4,5]. Ethiopia has one of the top maternal mortality ratios globally (MMR) with 412 maternal deaths per 100,000 live births. The frequency of these deaths drops with increasing periods from birth [6,7]. The majority of maternal and infant mortality takes place in the first month after birth. Practically half of post-natal maternal deaths take place within the first 24 h, 66 % occur during the first week after delivery, and one million newborns die on the first day of life. The key explanations for these avoidable problems were deprived excellence of services, fragile community-based health practice, gender discrimination, and deprived women-centered maternity care [8,9]. Ethiopia had a strategy to decline the MMR from 420 to 199/100,000 and the neonatal mortality rate of 29 to 10/1000 live births between 2015/16–12019/20 to accomplish the SDG. Here is an inadequate study on the early utilization of post-natal care services and related factors in Ethiopia [10]. So it is vital to take proof about the Early Post-natal Care Services Utilization and Its Associated Factors among women in Ethiopia to deliver approvals around appropriate approaches to boost utilization and to advance maternal and neonatal outcomes. This review makes available evidence on the prevalence of early utilization of post-natal care services and associated factors among women in Ethiopia.

## 2 Methods

This systematic review and meta-analysis were shown based on the methodology of chosen reporting items for the Systematic Review and Meta-analysis (PRISMA) checklist [11]**.**

### 2.1 Study setting and data sources

Ethiopia is a country in the Horn of Africa with two city administrations and twelve administrative regional states. Search the Web of Sciences using the Pub Med database, PubMed/MED-LINE, CINAHL, Google Scholar, and Scholar. Unpublished studies from a few research centers and library sources were also revised.

Only works published in Ethiopia and sources in the English language are reviewed. All of the listed electronic databases were recognized as sources for job seeking. This review contained ten studies in total.

**Fig. 1:**
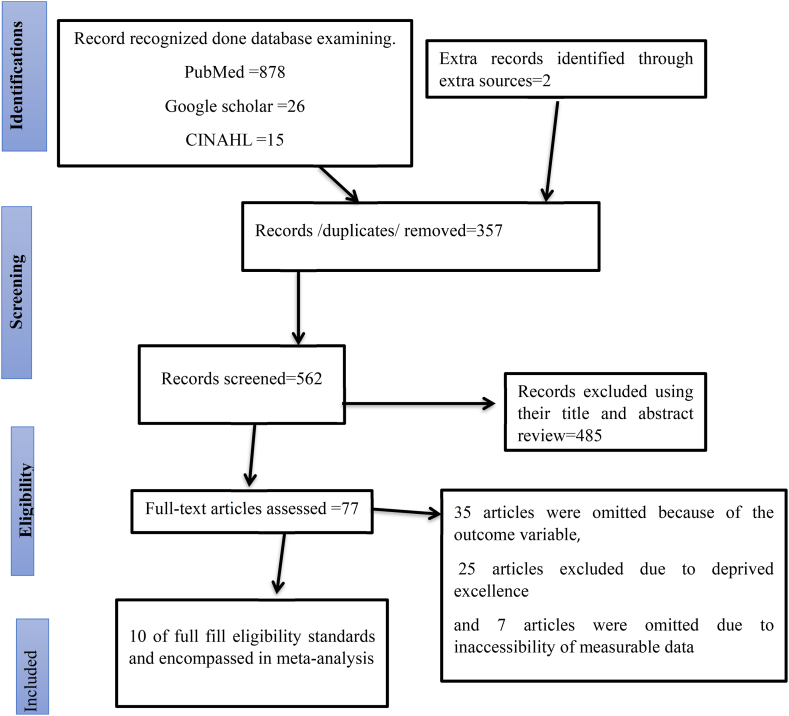
PRISMA follows the map of the examination and choice of studies for systematic review and meta-analysis of Early Post-natal Care Services Utilization and Its Associated Factors among Mothers in Ethiopia, 2023.

Six authors (TS, FA, HG, GN, EH, and WY) used inclusive searching algorithms to look through the studies. First, full titles (“Prevalence and Factors affecting utilization of early post-natal care services among mothers in Ethiopia”) were looked up to examine the articles, followed by keywords (“early post-natal care service,” “prevalence,” “utilization,” “associated factors” or “determinants,” and “Ethiopia”). Additionally, research was looked up from the reference lists of all included studies to locate other studies that were not found using our search strategies (Fig. 1).

### 2.2 Operational definition

**Utilization of early post-natal care services**: Mother who had at least one post-natal care check-up within 2–7 days.

### 2.3 Eligibility criteria

Published and unpublished studies conducted in Ethiopia between 2015 and May 2023 are included in the current systematic review, along with English-language articles.

The condition, context, and population method, or CoCoPoP, was utilized to specify the inclusion and exclusion criteria.

### 2.4 Inclusion

Research revealed how common early postpartum care services are in Ethiopia, as well as the factors that influence their use. Additionally, this review included all full-text articles with a response rate of at least 85 % that were authored in English.

## 3 Exclusion

Qualitative research and studies with insufficient data were excluded.

### 3.1 Study selection

With data-driven searching, 921 primary studies were identified. To get rid of duplicate research, all retrieved studies were moved to Endnote version ix. There were two phases involved in the selection of studies. Full-text review came after the selection of the title and abstract. Studies that indicated prevalence and factors influencing the utilization of early postnatal care contact mothers were carefully selected through title and abstract selection by all independent researchers. An article that was considered potentially appropriate by one reviewer was evaluated as a whole text and chosen by each reviewer separately after a full-text review. Ten research were included in the total; 485 studies were excluded because their title and abstract were incomplete, 357 studies were eliminated because they were duplicates, and 67 studies were rejected because they lacked sufficient quality or were not able to get quantifiable data.

### 3.2 Data extraction

A standardized generalization form created in an Excel spreadsheet was used to extract data. The following information was extracted for each study: the study region, study period, study design (prevalence of early postnatal care services), factors associated with early postnatal care services, odds ratio with 95 % confidence interval for early postnatal care services, and the total number of sample sizes included in the study. Identification data (first author last name and publication year) were also extracted for each study. After completing the data extraction for all specified study components, the researchers cross-checked the extracted data to look for any discrepancies. During the process of critical appraisal and data extraction, disparities among the writers were resolved with the involvement of a second author.

### 3.3 Quality of individual studies

The individual study's technical strength and quality were evaluated using the Newcastle-Ottawa Scale quality assessment instrument, which was applied to the original cross-sectional study used for cross-sectional study quality assessments [12]. The instrument has three fundamental mechanisms; the instrument's key component is categorized from five stars and mostly emphasizes the methodological quality of each primary study. The second mechanism of the instrument weighs up the equivalency of the main studies involved in this systematic review and meta-analysis. The instrument's last component measured the quality of the main articles in statistical analysis.

The qualities of the individual original study were weighted by six authors individually using these pointers. Studies with a medium score (satisfying 50 % quality evaluation criteria) and high quality ((≥7 out of 10) were registered for analysis, the five investigators' differences were managed by taking the average score of their quality evaluation outcomes **(Additional file 1).**

**Outcome of interest:** Early postnatal care services' utilization prevalence was the primary outcome examined in this systematic review and meta-analysis. The review's second goal was to identify the variables influencing the prevalence.

### 3.4 Publication bias and heterogeneity

Electronic database examinations have been used to reduce the risk of bias. The authors' supportive work, selection of articles based on clear objectives, and eligibility criteria, determining the study's quality, and extracting and compiling the data were also important in decreasing bias. We observe publication bias with a visual inspection of the funnel plot graph qualitatively suggests asymmetry (Fig. 4). Moreover, Egger's correlation tests at a 5 % significant level showed the presence of publication bias (Table 2). In addition, to diminish the random variations among the primary study's point estimates, subgroup analysis was done by study regions. Sensitivity analysis was also done to identify the possible source of heterogeneity. Heterogeneity through studies was estimated using inverse variance (I^2^) statistics with its corresponding p-value using the random-effect model.

**Table 1:** Summary of the 10 observational studies included in the meta-analysis Factors affecting utilization of early postnatal care services among mothers in Ethiopia, 2023.

Authors	Study period	Study Setting	Study population	Sample size	Response rate (%)	Prevalence	Title of study's
Dona.A et al.	2022	Community-based	Women	306	99.5	66.7	Factors influencing utilization of early postnatal care services.
Gebre. G et al.	2019	Community-based	Women	395	92.7	22.5	Early postnatal care service utilization and associated factors.
Tefera. Y et al.	2021	Community-based	Women	612	100	13.7	Early Postnatal Care Service Utilization and Its Determinants.
Yosef. Y et al.	2023	Community-based	Women	306	98.4	23.3	Prevalence of early postnatal care Services usage and associated factors.
AN.T et al.	2017	Community-based	Women	382	99.2	23.7	Early Postnatal Care Service Utilization and Associated Factors.
Alemu.T et al.	2021	Community-based	Women	612	98.5	24.9	Early Postnatal Care Service Utilization and Associated Factors.
Gebreslassie.T et al.	2018	Community-based	Women	481	99.4	34.3	Prevalence and Associated Factors of Early Postnatal Care Service.
Yoseph.S et al.	2021	Community-based	Women	320	100	29.7	Early Postnatal Care Service Utilization and Associated Factors.
Ayele. L et al.	2022	Community-based	Women	752	93	21.8	Early Postnatal Care Utilization Service Utilization.
Melesse.T	2020	Community-based	Women	332	100	25.5	Postnatal Care within One Week and Associated Factors.

**Table 2:**
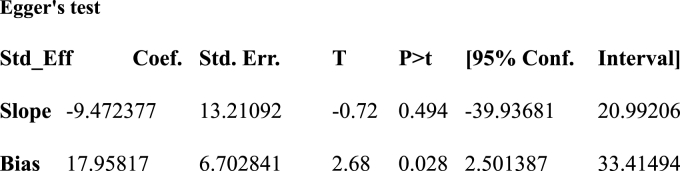
Eggers statically test to check the Publication bias among the ten studies among women in Ethiopia, 2023.

### 3.5 Statistical analysis

We used Microsoft Excel for data entry and STATA version 17 software to analyze the prevalence of early post-natal care services and associated factors. Random effects model based on the DerSimonian-Laird technique was measured to measure differences between the studies, to minimize the variance of estimated points between primary studies, a sub-group analysis was approved about the regions. The results were presented using texts, tables, and forest plots with measures of effect and a 95 % confidence interval. Publication bias (small study effect) was checked using graphical and Eggers statistical tests. The statistically significant Eggers test (*P*-value <0.05) indicates the presentence of a small study effect and is handled by non-parametric trim and fill analysis using the random effects model [13].

## 4 Result

### 4.1 Description of studies

A total of 921 primary studies were recognized. Out of these 357 studies were omitted due to duplications, 485 studies were omitted due to their title and abstract were not full and 67 studies were omitted due to deprived quality and inaccessible measurable data only 10 studies were fulfilled the inclusion criteria and were included in this systematic review and meta-analysis with a total population of 4498 women (Fig. 1).

### 4.2 Characteristics of the included studies

All 10 eligible studies were Community-based cross-sectional in design and were reported in the English language. The sample size ranged from 306 women [14] to 752 in Oromiya. Regarding the geographical distribution of studies, three studies were from Oromiya Two from Sidama [14,15], (one from Tigray [16], and four from Refs. [[17], [18], [19]]SNNP. all included studies reported on the prevalence of Early Post-natal Care Services Utilization and factors associated with Early Post-natal Care Services Utilization **(**Table 1**).** Out of the total 10 studies, eight had a quality score of eight and the remaining two had a quality score of seven (**Additional file 2**).

### 4.3 Pooled prevalence of early post-natal care services utilization in Ethiopia

**Fig. 2:**
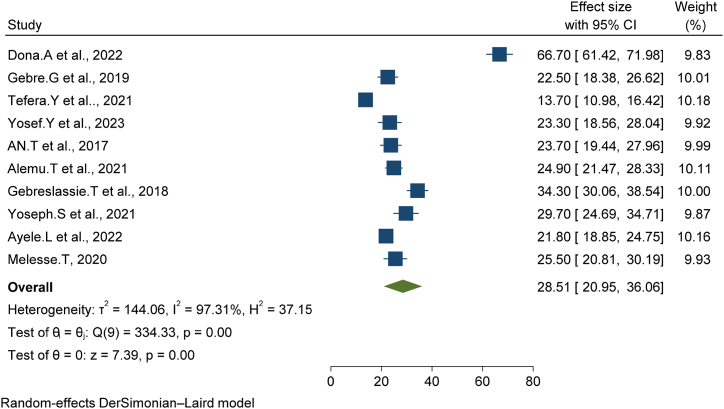
Forest plot of the pooled prevalence of early postnatal care services utilization in Ethiopia, 2023.

The overall pooled prevalence of early postnatal care services utilization in Ethiopia was (28.51 % (95%CI, [20.95, 36.06]) (Fig. 2). The highest weight among the studies observed from studies conducted by Donal. A et al. [14].

### 4.4 Sensitivity analysis

**Fig. 3:**
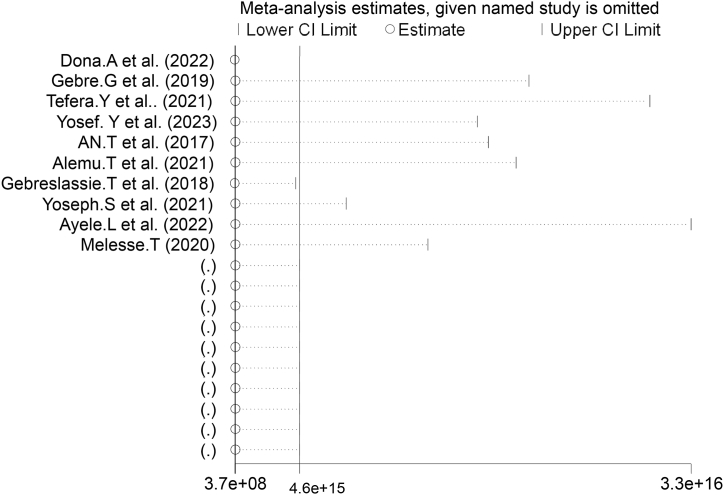
Funnel plot to test the publication bias of ten studies.

To discover outliers of a single study's influence on the overall meta-analysis outcome. Sensitivity analysis was conducted using a random-effects model the result revealed no clear suggestion for the effect of a single study on the overall meta-analysis result (Fig. 3).

**Fig. 4:**
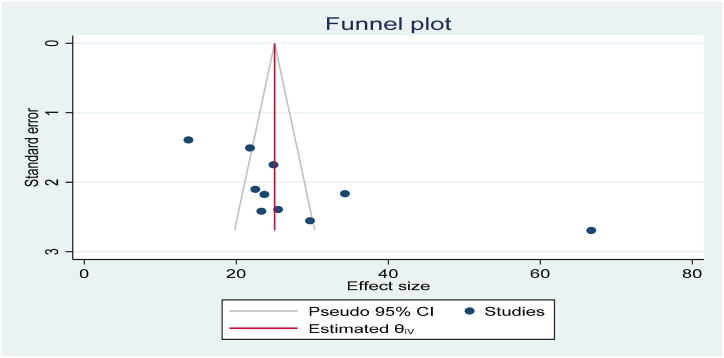
Subgroup analysis based on the administrative region of the country among women in Ethiopia, 2023.

### 4.5 Heterogeneity

Between the ten studies from the random effects model pooled estimate significant heterogeneity was observed (I –squired = 97.31, p = 0.000≤).

### 4.6 Publication bias

Funnel plot and Eggers statically test were done to check the Publication bias among the ten studies. The funnel plot showed an asymmetric shape, which shows the presence of publication bias (Fig. 4). Eggers statically test verified the presence of publication bias between the studies (β = 2.68 p-values = 0.028) (Table 2).

### 4.7 Subgroup analysis

**Fig. 5:**
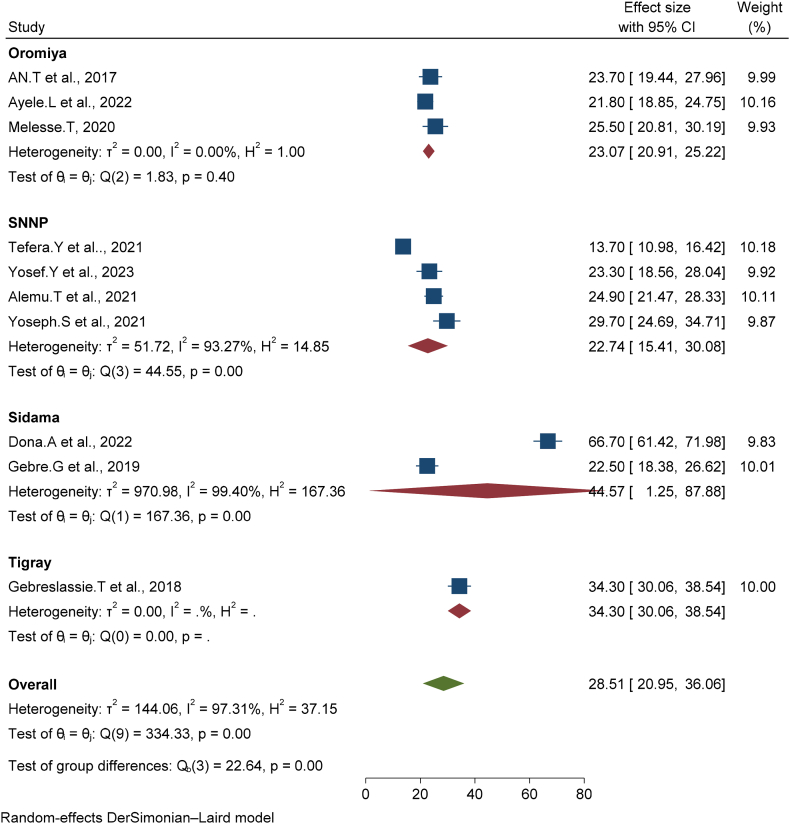
Factors affecting the prevalence of Early Post-natal Care Services Utilization among women in Ethiopia, 2023. (Analysis by random model).

Subgroup analysis was conducted through the administrative regions of the country and the highest pooled effect of prevalence of early postnatal care services utilization was seen in the Sidama region at 44.57 % [95%CI,1.25,87.88] followed by Tigray at 34.30 % [95%CI,30.06,38.4]. Whereas the least pooled prevalence effect was detected in the SNNP region with a pooled prevalence of 22.74 % (95%CI15.41, 30.08) (Fig. 5).

### 4.8 Factor associated with early post-natal care services utilization

**Fig. 6:**
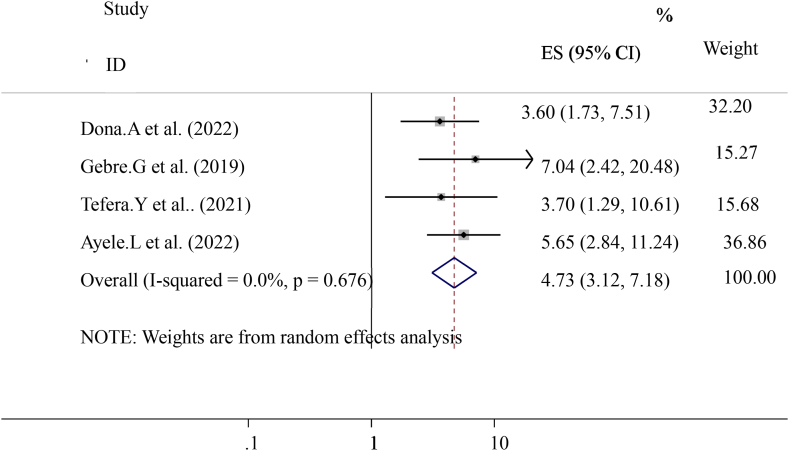
Forest plot showing pooled odds ratio for the association between having formal education with the prevalence of early post-natal care services utilization among women in Ethiopia, 2023.

This systematic review and meta-analysis discovered that the prevalence of early post-natal care services utilization in Ethiopia was significantly associated with educational status (having formal education), knowledge about early post-natal care visits, birth complication, institutional delivery, history of ANC follow-up distance from the health center, women's decision-making empowerment and previous history of early post-natal care visit were the factors. According to this study, the pooled effect of four primary studies identified that having formal education was significantly associated with the prevalence of early post-natal care services utilization ([14,16,19,20]). Women who have had formal education were four times more likely to have early post-natal care services utilization than women who had no history of formal education ((OR = 4.73 (95%CI, 3.12, 7.18)). The heterogeneity test indicated I^2^ = 0.0 %, *P = 0.676*. The random-effect model was applicable for analysis (Fig. 6).

**Fig. 7:**
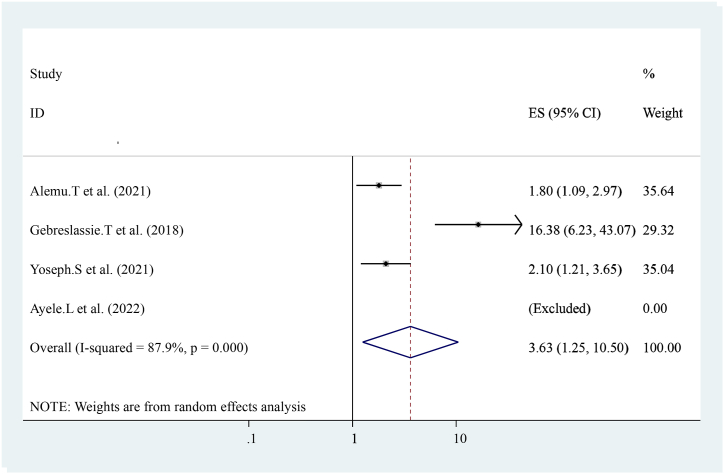
Forest plot showing pooled odds ratio for the association between Knowledge about early post-partum care visits with the prevalence of early post-natal care services utilization among women in Ethiopia, 2023.

The pooled effect of three studies reported that Knowledge about early post-natal care visits is strongly associated with early post-natal care services utilization [16,17,20,21] compared with others who have not. Women who had Knowledge about early post-natal care visits were three times more likely to have early post-natal care services utilization ((OR = 3.63 (95%CI, 1.25, 10.50)). The heterogeneity test indicated I^2^ = 87.9 %, P = 0.000. The random-effect model was applicable for analysis (Fig. 7).

**Fig. 8:**
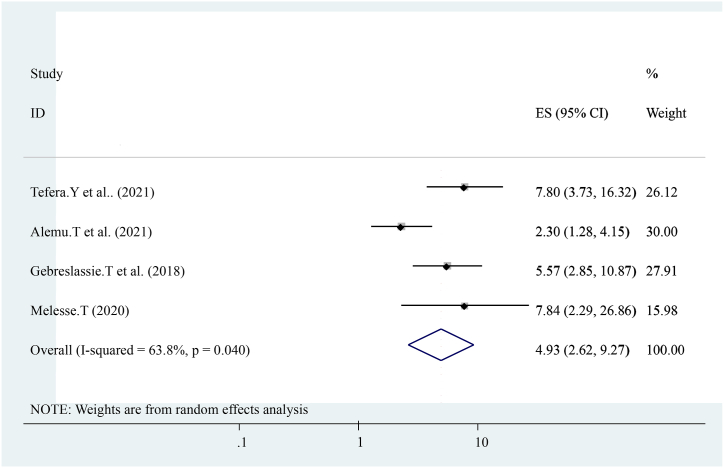
Forest plot showing pooled odds ratio for the association between Birth complications with the prevalence of early post-natal care services utilization among women in Ethiopia, 2023.

The pooled effect of four studies reported that Birth complications were strongly associated with early post-natal care services utilization [16,19,21,22]compared with others who have not. Women who had Birth complications during early post-natal care visits were five times more likely to have early post-natal care services utilization ((OR = 4.93 (95%CI, 2.62, 9.27)). The heterogeneity test indicated I^2^ = 63.8 %, P = 0.040. The random-effect model was applicable for analysis (Fig. 8).

**Fig. 9:**
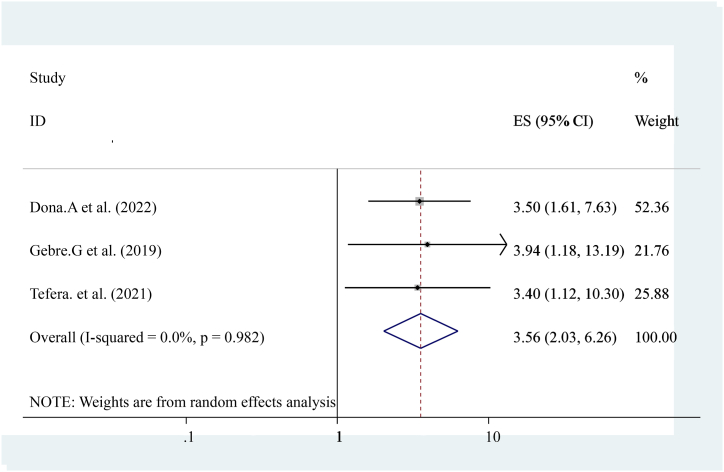
Forest plot showing pooled odds ratio for the association between ANC attendances with the prevalence of early post-natal care services utilization among women in Ethiopia, 2023.

The pooled effect of three studies reported that having ANC attendance is strongly associated with early postnatal care services utilization [14,16,19] compared to others who have not. Women who had had ANC care were Three times more likely to have early post-natal care services utilization ((OR = 3.56 (95%CI, 2.03, 6.26)). The heterogeneity test indicated I^2^ = 0.0 %, P = 0.982 hence the random-effect model was applicable for analysis (Fig. 9).

**Fig. 10:**
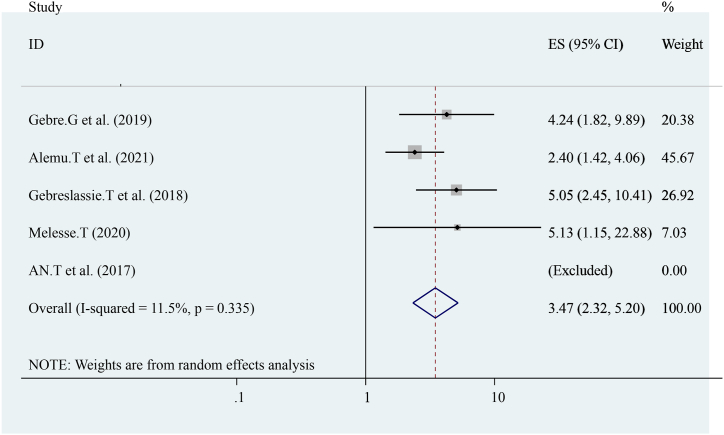
Forest plot showing pooled odds ratio for the association between distances from health centers with the prevalence of early post-natal care services utilization among women in Ethiopia, 2023.

The pooled effect of four studies reported that distances from health centers were strongly associated with early postnatal care services utilization [15,16,21,22]compared to others. Women who had traveled less than 2 h were Three times more likely to have early postnatal care services utilization ((OR = 3.47 (95%CI, 2.32, 5.20)). The heterogeneity test indicated I^2^ = 11.5 %, P = 0.335 hence the random-effect model was applicable for analysis (Fig. 10).

**Fig. 11:**
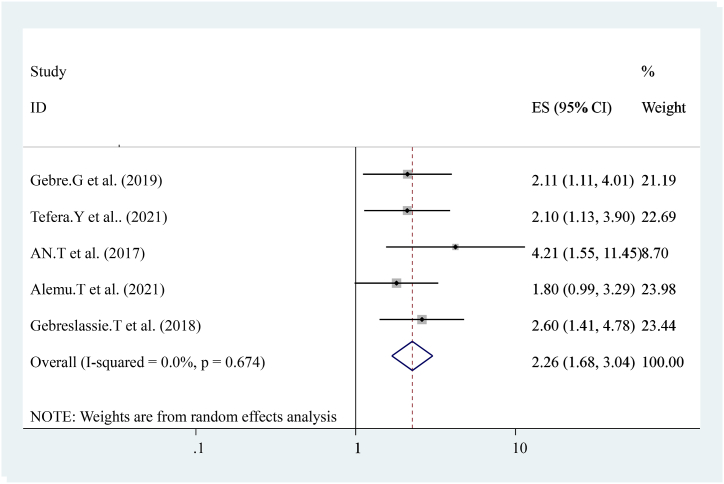
Forest plot showing pooled odds ratio for the association between previous histories of early post-natal care services utilization with the prevalence of early post-natal care services utilization among women in Ethiopia, 2023.

The pooled effect of five studies [15,16,19,21]also reported that previous history of early post-natal care services utilization was strongly associated with current early post-natal care services utilization compared to others. Women who had a history of early post-natal care services utilization were Two times more likely to have early post-natal care services utilization ((OR = 2.26 (95%CI, 1.68, 3.04)). The heterogeneity test indicated I^2^ = 0.0 %, P = 0.674 hence the random-effect model was applicable for analysis (Fig. 11).

**Fig. 12:**
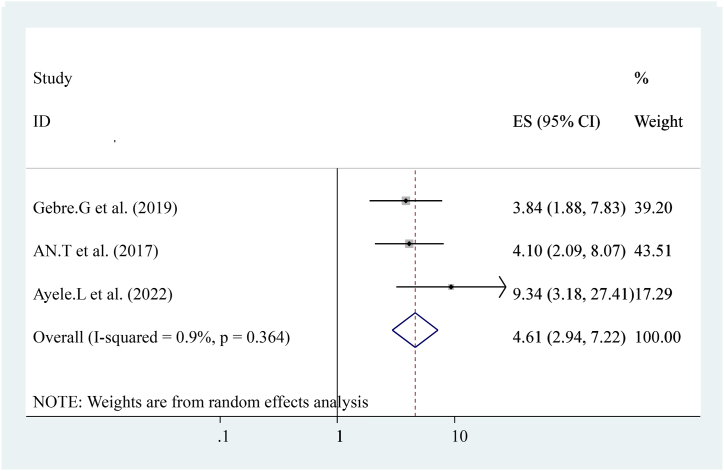
Forest plot showing pooled odds ratio for the association between women's empowerment with the prevalence of early post-natal care services utilization among women in Ethiopia, 2023.

The pooled effect of three studies reported that women's empowerment [15,20]. Was strongly associated with early post-natal care services utilization women who make decisions by themselves were four times more likely to have early post-natal care services utilization compared to others ((OR = 4.61 (95%CI, 2.94, 7.22)). The heterogeneity test indicated I^2^ = 0.9 %, P = 0.364. The random-effect model was applicable for analysis (Fig. 12).

**Fig. 13:**
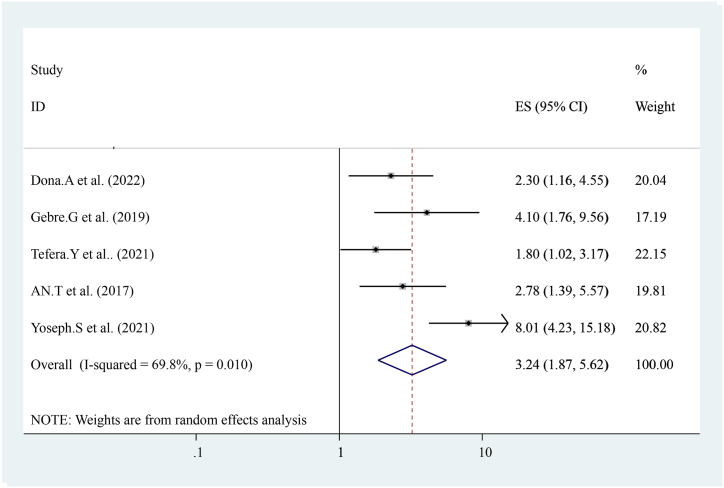
Forest plot showing pooled odds ratio for the association between institutional deliveries with the prevalence of early post-natal care services utilization among women in Ethiopia, 2023.

The pooled effect of five studies [14,15,17,19]reported that institutional delivery is strongly associated with early post-natal care services utilization compared to others. Women who had a history of institutional delivery were three times more likely to have early post-natal care services utilization ((OR = 3.24 (95%CI, 1.87, 5.62)). The heterogeneity test indicated I^2^ = 69.8 %, P = 0.010 hence the random-effect model was applicable for analysis (Fig. 13).

## 5 Discussion

The pooled prevalence of early postnatal care services utilization in Ethiopia was low related to different studies, and the national and WHO recommendations. This systematic review and meta-analysis aimed to assess the pooled prevalence of early postnatal care services utilization and associated factors in Ethiopia. This meta-analysis is the principal of its type in defining the national coverage and associated factors of early postnatal care services utilization in Ethiopia. Evaluating the national level and its factors allows legislators, the Ethiopian Ministry of Health as well as different stakeholders, NGOs, future researchers clinicians, or health care providers to improve evidence-based strategies and serve as input at a national level for the improvement of early postnatal care services utilization. The overall pooled prevalence of early postnatal care services utilization in Ethiopia was (28.51 % (95%CI, [20.95, 36.06]) this finding is lower than the EDHS 2019 report it was 34 % but higher than the 2016 EDHS finding [6].

The pooled prevalence of this finding is also lower than the 2016 Demographic and Health Survey in Uganda, Kenya, and Ghana that show the national prevalence of early postnatal care services utilization was 50 % 53 %, and 31 % respectively [[23], [24], [25]]. This difference might be due to variances in health system coverage or strategies and differences in socioeconomic status. In addition, diffidence might be the individual differences in health care providers, health facilities differences, and health system differences as to socioeconomic, cultural aspects, study time, data collection time, and sampling technique. In this study, different factors like educational status (having formal education), knowledge about early postnatal care visits, birth complication, institutional delivery, history of ANC follow-up distance from the health center, women's decision-making empowerment, and previous history of early post-natal care visit were statically and significantly associated with early post-natal care services utilization. From the pooled effect women who had formal education had four times (OR = 4.73 (95%CI, 3.12, 7.18)) and having ANC attendance ((OR = 3.56 (95%CI, 2.03, 6.26)) were three times more likely to have early post-natal care services utilization respectively. This finding is similar to the study conducted in Uganda. Distances from health center ((OR = 3.47 (95%CI, 2.32, 5.20)) women who had traveled less than 2 h were three times more likely to have early post-natal care services utilization. This finding is similar to the national finding in Uganda [24]. Knowledge about early post-natal care visit ((OR = 3.63 (95%CI, 1.25, and 10.50)) three times more likely to have early post-natal care services utilization. Birth complication ((OR = 4.93 (95%CI, 2.62, 9.27)) is five times more likely to have early post-natal care services utilization. Previous history of early post-natal care services ((OR = 2.26 (95%CI, 1.68, 3.04)) women who had a history of early post-natal care services utilization were two times more likely to have early post-natal care services utilization. This might be due to women getting counseling or advice from health care providers about early post-natal care services during the antenatal period. Women's empowerment ((OR = 4.61 (95%CI, 2.94, 7.22)) four times more likely to have early post-natal care services utilization and institutional Delivery ((OR = 3.24 (95%CI, 1.87, 5.62)) Women who had a history of institutional Delivery had three times more likely to have early post-natal care services utilization. This finding is also similar to the study conducted in Uganda. In conclusion, The pooled prevalence of Early Post-natal Care Services Utilization in Ethiopia shows low related to different studies, the WHO, and countrywide recommendations. Educational status (having formal education), knowledge about early post-natal care contact, birth complication, institutional delivery, history of ANC follow-up distance from the health center, women's decision-making empowerment, and previous history of early post-natal care contact were associated with Early Post-natal Care Services Utilization in Ethiopia. In addition, early postnatal care services utilization highly influenced by birth complication and antenatal care services utilization. Enhancing antenatal care services coverage at a national level is needed to improve early postnatal care services utilization. Emphasizing or improving the knowledge and attitudes of the mothers towards early post-natal care contact, boosting delivery at health facilities, and increasing the number of Health centers, and health posts are essential to improve quality of life and diminish neonatal and maternal morbidity and mortality.

### 5.1 Strengths and limitations of the study

There are some limitations near the strength of the national suggestion of this review about the pooled prevalence of early postnatal care services utilization in Ethiopia and associated factors, the main drawback was the lack of articles from different portions of the country in overall Ethiopia. But it provides data about the national status of early post-natal care services utilization and its predictors or factors at a national level this helps the federal Ministry of Health as an input.

There is also a lack of systematic reviews and meta-analysis articles throughout this making it challenging to associate this review with different studies.

## 6 Conclusion and recommendation

In comparison to national guidelines, the WHO, and other research, Ethiopia's pooled prevalence of accessing early postnatal care services is low. Prenatal care service use and birth complications also have a significant impact on the use of early postnatal care services. Improving early postnatal care service usage requires expanding the availability of antenatal care services on a national scale. Strengthening prenatal care services, increasing the number of health centers and health posts, increasing delivery at health facilities, and emphasizing or improving mothers' knowledge of and attitudes toward early post-natal care contact are all critical to improving quality of life and lowering neonatal and maternal morbidity and mortality. Future studies and the Ethiopian Ministry of Health should concentrate on improving the use of prenatal care services, minimizing and managing birth complications, and enhancing the use of early postnatal care services.

## Data availability

Data will be made available on a reasonable request.

## Funding

This research received no specific funding from any funding agency in the public, profitable, or not-for-profit sectors.

## Informed consent

Informed consent was not required for this article since we are using a primary study.

## Ethical approval

Ethical approval was not required for this article since the study was shown based on data extracted from published studies.

## Contributor ship

TS, FA, EH, GN, and WY explored the literature and considered the study. HG was involved in procedure development and data analysis. FA wrote the first draft of the manuscript. All authors reviewed and edited the document and accepted the final version of the manuscript.

## CRediT authorship contribution statement

**Tigist Seid Yimer:** Writing – review & editing, Writing – original draft, Visualization, Validation, Supervision, Software, Resources, Project administration, Methodology, Investigation, Funding acquisition, Formal analysis, Data curation, Conceptualization. **Florence Ayalew Sisay:** Supervision, Software, Investigation, Formal analysis, Data curation, Conceptualization. **Habtamu Gebrehana Belay:** Funding acquisition, Formal analysis, Data curation, Conceptualization. **Gedefaye Nibret Mihretie:** Writing – original draft, Visualization, Validation, Supervision, Software, Resources. **Eyaya Habtie Dagnaw:** Visualization, Validation, Supervision, Software, Project administration, Investigation, Data curation. **Wassie Yazie Ferede:** Writing – review & editing, Validation, Supervision, Methodology, Funding acquisition, Formal analysis.

## Declaration of competing interest

The authors declare that they have no known competing financial interests or personal relationships that could have appeared to influence the work reported in this paper.
