# Supplementary material for: Early Post-natal Care Services Utilization and its associated factors among mothers Systemic Review and Meta-Analysis
Source: Heliyon. 2023 Dec 18;10(1):e23760. doi: 10.1016/j.heliyon.2023.e23760 (PMC10772637; doi:10.1016/j.heliyon.2023.e23760)
Supplement: Multimedia component 1 [file mmc1.docx]

**Additional file 1:** Searching strategy for Early Post-natal Care Services Utilization and Its Associated Factors among Mothers in Ethiopia, 2023 a systematic review and meta-analysis

| Databases | Searching terms | Number of studies |
| --- | --- | --- |
| PubMed | (("epidemiology"[Subheading] OR "epidemiology"[All Fields] OR "prevalence"[All Fields] OR "prevalence"[MeSH Terms]) AND Factors[All Fields] AND affecting[All Fields] OR "utilization"[All Fields]) AND early[All Fields] AND ("post-natal care"[MeSH Terms] OR ("post-natal"[All Fields] AND "care"[All Fields]) OR "post-natal care"[All Fields] OR ("post"[All Fields] AND "natal"[All Fields] AND "care"[All Fields]) OR "post-natal care"[All Fields]) AND "services"[All Fields] AND ("mothers"[MeSH Terms] OR "mothers"[All Fields]) AND ("Ethiopia"[MeSH Terms] OR "Ethiopia"[All Fields])). | 878 |
| Google scholar | Prevalence and predictors of Early Post-natal Care Services Utilization and Its Associated Factors in Ethiopia | 26 |
| CINAHL | Prevalence and predictors of Early Post-natal Care Services Utilization and Its Associated Factors in Ethiopia | 15 |
| Others databases |  | 2 |
| Total retrieved |  | 921 |
| Included |  | 10 |
